# Supplementary material for: Reinforced Nafion Membrane with Ultrathin MWCNTs/Ceria Layers for Durable Proton-Exchange Membrane Fuel Cells
Source: Membranes (Basel). 2022 Oct 29;12(11):1073. doi: 10.3390/membranes12111073 (PMC9698217; doi:10.3390/membranes12111073)
Supplement: Supplementary file 1 [file membranes-12-01073-s001.zip › membranes-1972867-supplementary.pdf]

membranes

MDPI

Supplementary Materials: Reinforced Nafion Membrane with Ultrathin MWCNTs/Ceria Layers for Durable Proton-Exchange Membrane Fuel Cells

Dongsu Kim [1,†], Yeonghwan Jang [1,†], Eunho Choi [1], Ji Eon Chae [2], and Segeun Jang [1,*]

[1] School of Mechanical Engineering, Kookmin University, Seoul 02707, Korea
[2] Department of Mobility Power Research, Korea Institute of Machinery & Materials, 156 Gajeongbuk-ro, Yuseong-gu, Daejeon 34103, Korea
[*] Correspondence: sjang@kookmin.ac.kr
[†] These authors contributed equally to this work.

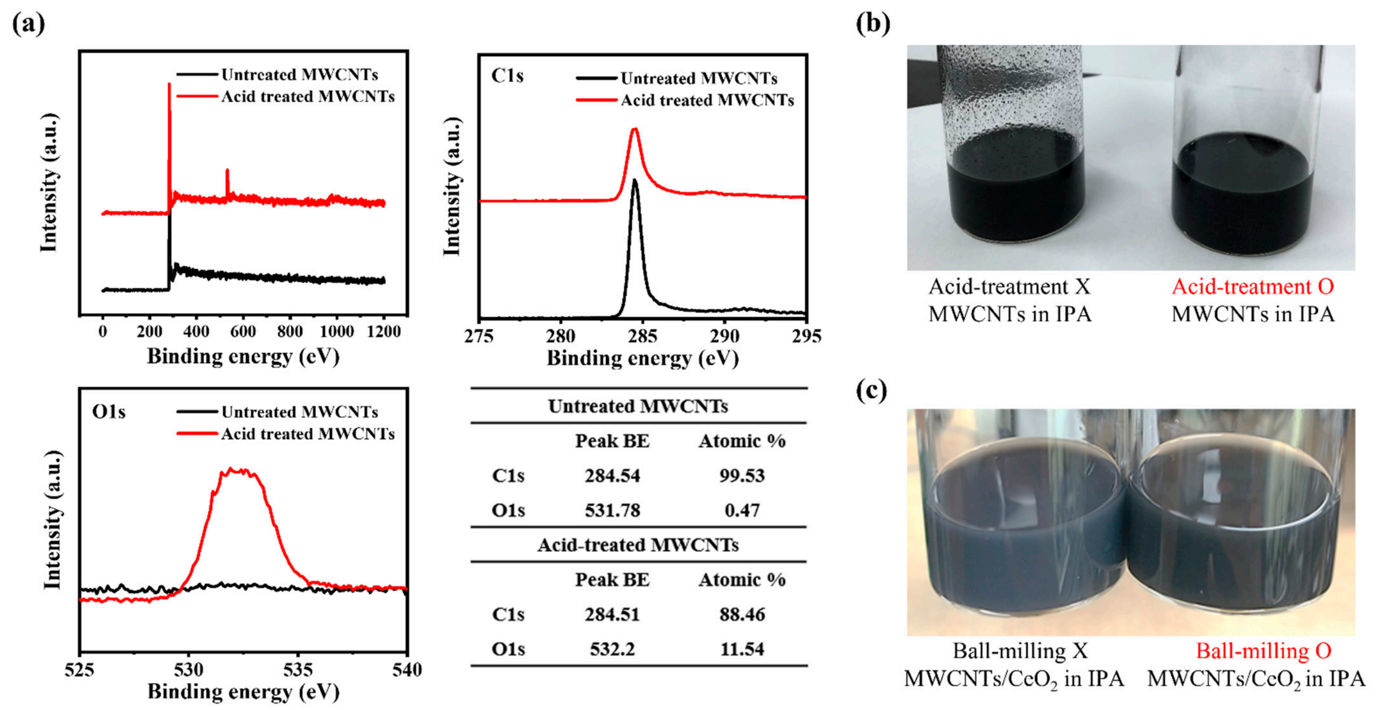

Figure S1. (a) XPS spectra of acid-treated and untreated MWCNTs. (b) The difference in the dispersion of MWCNTs with or without acid treatment. (c) The difference in the dispersion of MWCNTs/CeO_2 with or without ball-mill.

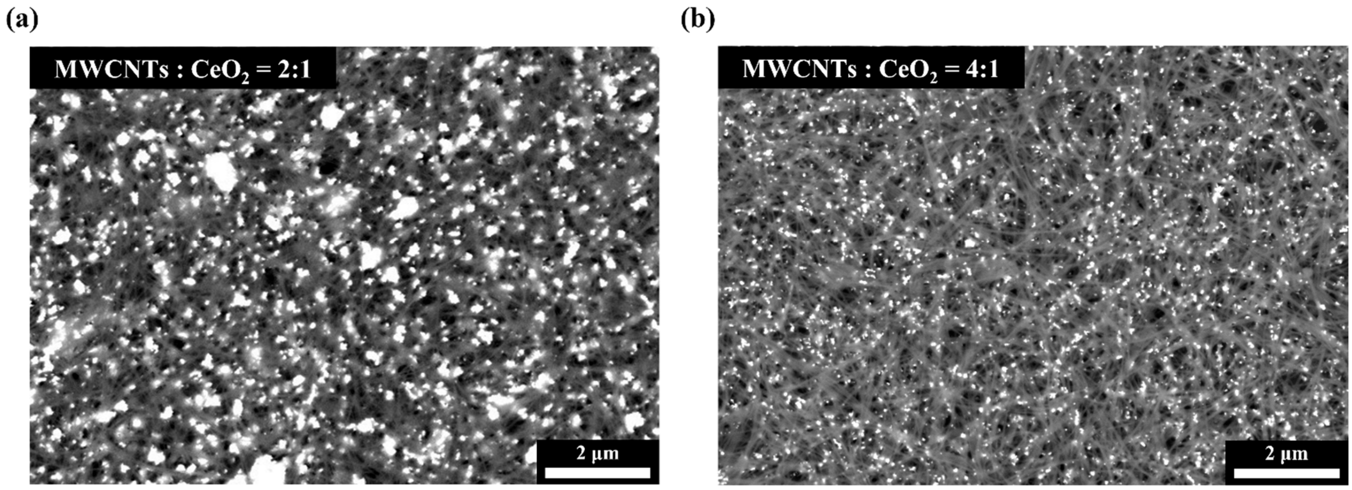

Figure S2. SEM surface images of (a) MWCNTs:CeO_2=2:1 and (b) MWCNTs:CeO_2=4:1

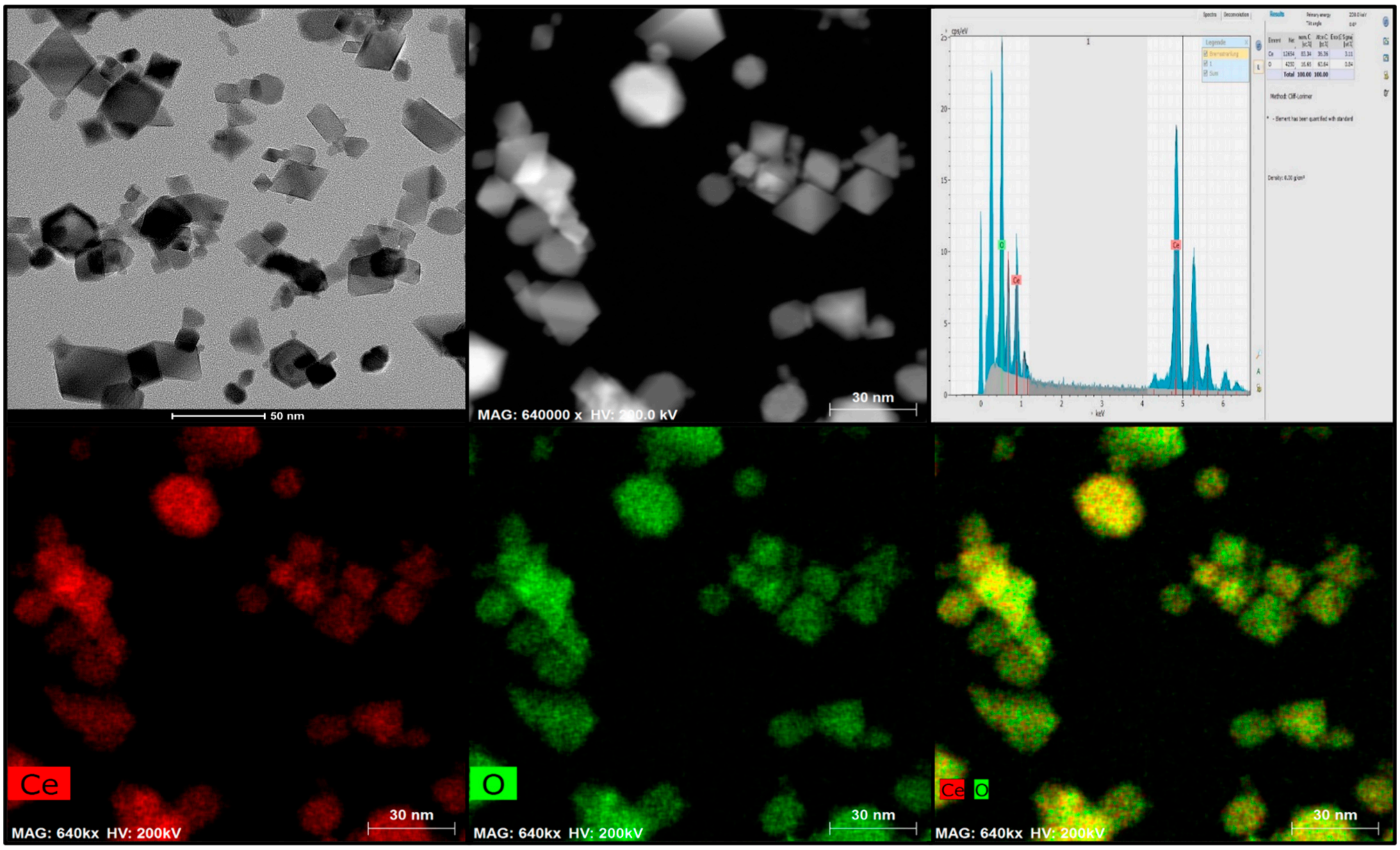

Figure S3. TEM images and corresponding EDX analysis of CeO_2 nanoparticles.

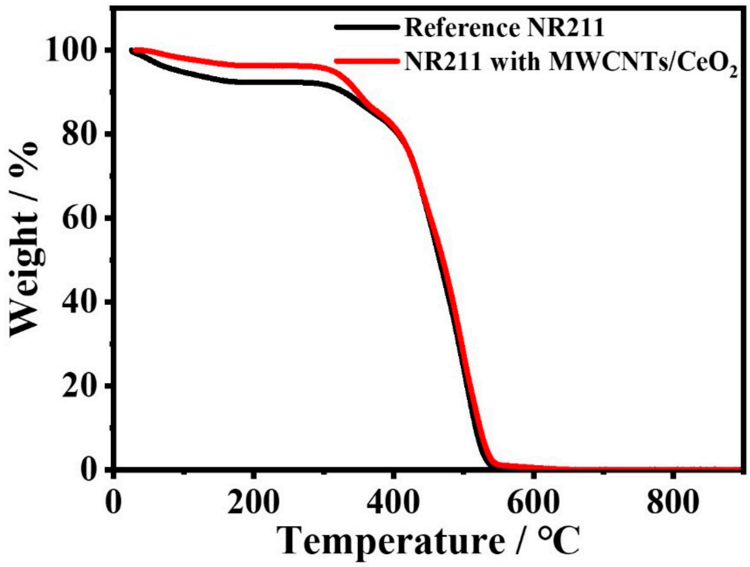

Figure S4. TGA curves of NR211 with MWCNTs/CeO_2 membrane and reference NR211 membrane.

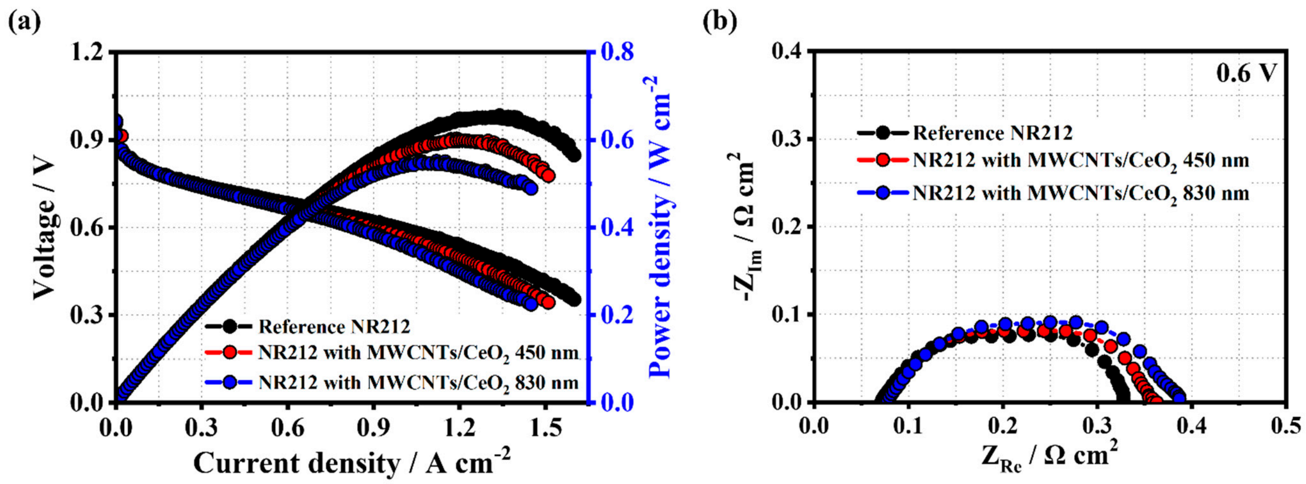

Figure S5. (a) Polarization curves and (b) corresponding EIS spectra at 0.6V for MEAs with reference NR212, NR212 with MWCNTs/CeO_2 layers 450 nm, and NR212 with MWCNTs/CeO_2 layers 830 nm.

Table S1. Summary of key parameter values

Sample	Peak power density [W cm^{-2}]	Current density [A cm^{-2}]	R_{ohm} [Ω cm^2]	R_{ct} [Ω cm^2]
**Reference NR212**	0.655 (-)	0.98 (-)	0.0713 (-)	0.2565 (-)
**NR212 with MWCNTs/CeO_2 450 nm**	0.602 (-8.10%)	0.90 (-8.17%)	0.0742 (+3.91%)	0.2842 (+9.75%)
**NR212 with MWCNTs/CeO_2 830 nm**	0.550 (-16.04%)	0.83 (-15.31%)	0.0771 (+7.53%)	0.3095 (+17.13%)
